# Supplementary material for: Nanoscale Functional Additives Application in the Low Temperature Greases
Source: Polymers (Basel). 2021 Oct 29;13(21):3749. doi: 10.3390/polym13213749 (PMC8586967; doi:10.3390/polym13213749)
Supplement: Supplementary file 1 [file polymers-13-03749-s001.zip › polymers-1332512-Suplementarry.pdf]

Supporting info

Table S1. Study of the NFA Impact on the Physicochemical and Antiwear and Antiscuffing Properties of Complex Lithium Greases.

**No.**	**Name of indicator**	**Base grease**	**Nano-functional additive**											
			**Montmorillonite K 10**		**Silicon dioxide (KOVELOS 35/01T)**		**Calcium Carbonate ($CaCO_3$)**		**Calcium borate**		**Halloysite**		**MoS_2 on halloysite**	
			**The method of additive addition (1 - before heat treatment, 2 - after heat treatment)**											
			**1**	**2**	1	**2**	**1**	**2**	**1**	**2**	**1**	**2**	**1**	**2**
**1**	Yield stress at 50°C, Pa	**680**	540	560	660	590	660	380	120	590	730	70 0	760	710
**2**	Colloidal stability,% of oil emitted	**19.6**	20.9	18.8	19.4	21.0	23.7	24.7	35.2	19.9	18.9	24.7	19.7	24.0
**3**	Dropping point, ° C	**> 250**	240	234	> 250	234	> 250	230	222	> 250	245	211	2 44	226
4	Effective viscosity at minus 50ºC	**1331**	1382	1263	1384	1245	1274	1274	450	1211	1161	1186	1211	1191
**5**	Lubricating properties on a four-ball friction machine under the temperature of (20 ± 5) °C:													
	- welding load, kgs	**178**	224	200	237	237	188	200	224	237	200	211	200	200
	- wear scar, mm	**0.40**	0.76	0.64	0.36	0.39	0.38	0.35	0.36	0.35	0.48	0.48	0.41	0.42

Table S2. Study of the NFA Impact on the Physicochemical and Antiwear and Antiscuffing Properties of Polyurea Greases.

**No.**	**Name of indicator**	**Base grease**	**Nano-functional additive**											
			Montmorillonite K 10		Silicon dioxide (KOVELOS 35/01T)		Calcium Carbonate ($CaCO_3$)		Calcium borate		Halloysite		MoS_2 on halloysite	
			**The method of additive addition (1 - before heat treatment, 2 - after heat treatment)**											
			**1**	**2**	**1**	**2**	**1**	**2**	**1**	**2**	**1**	**2**	**1**	**2**
**1**	Yield stress at 50°C, Pa	**260**	340	240	360	310	4 60	320	3 60	260	370	260	390	200
**2**	Colloidal stability,% of oil emitted	**24.2**	17.7	19.5	18.3	21.6	16.3	19.8	18.7	21.2	20.7	24.0	16.2	24.1
**3**	Dropping point, ° C	**2 21**	> 250	219	> 250	230	> 250	230	> 250	227	238	22 5	233	223
**4**	Effective viscosity at minus 50ºC	**928**	1409	1081	1354	1105	1324	1081	1 379	1185	1669	963	1779	820
**5**	Lubricating properties on a four-ball friction machine under the temperature of (20 ± 5)°C:													
	- welding load, kgs	**112**	119	126	133	126	211	200	160	160	141	141	224	224
	- wear scar, mm	**0.69**	0.73	0.75	0.56	0.58	0.72	0.72	0.61	0.72	0.49	0.57	0.66	0.79

Table S3. Study of the NFA Impact on the Physicochemical and Antiwear and Antiscuffing Properties of Polymer Greases.

No.	Name of indicator	Base grease	Nano-functional additive											
			Montmorillonite K 10		Silicon dioxide (KOVELOS 35/01T)		Calcium Carbonate ($CaCO_3$)		Calcium borate		Halloysite		MoS_2 on halloysite	
			The method of additive addition (1 - before heat treatment, 2 - after heat treatment)											
			1	2	1	2	1	2	1	2	1	2	1	2
1	Yield stress at 50°C, Pa	**140**	360	380	300	130	50	120	110	120	330	100	400	120
2	Colloidal stability,% of oil emitted	**25.7**	24.0	23.8	20.1	26.5	19.7	20.1	20.2	20.8	22.8	25.4	21.2	26.1
3	Dropping point, ° C	**145**	143	149	143	150	143	144	144	145	143	1 46	142	144
4	Effective viscosity at minus 50ºC	**2482**	3123	3240	1 849	2120	2544	2389	2 3 76	2417	2022	2135	2127	2182
5	Lubricating properties on a four-ball friction machine under the temperature of (20 ± 5)°C:													
	- welding load, kgs	**160**	160	160	160	16 0	168	211	168	160	160	178	178	188
	- the wear scar, mm	**0.87**	0.88	0.90	0.89	0.88	0.87	0.88	0.84	0.88	0.88	0.92	0.70	0.54

Table S4. Study of the NFA Impact on the Physicochemical and Antiwear and Antiscuffing Properties of Complex Lithium Greases.

No.	Name of indicator	Base grease	Nano-functional additive								
			Amorphous silicon dioxide (KOVELOS 35/01T)			Calcium Carbonate ($CaCO_3$)			Calcium borate		
			The NFA concentration, %								
			1%	2%	3%	1%	2%	3%	1%	2%	3%
1	**Yield stress at 50°C, Pa**	**680**	660	690	900	660	700	740	590	400	140
2	Colloidal stability,% of oil emitted	**19.6**	19.4	18.1	17.3	23.7	20.7	15.3	19.9	26.0	30,0
3	Dropping point, ° C	**> 250**	> 250	> 250	> 250	> 250	> 250	> 250	> 250	> 250	> 250
4	Effective viscosity at minus 50ºC	**1331**	1384	1565	1630	1274	1476	2192	1211	1184	1035
5	Lubricating properties on a four-ball friction machine under the temperature of (20 ± 5)°C:										
	- welding load, kgs	**178**	237	237	237	188	200	200	237	237	237
	- the wear scar, mm	**0.40**	0.36	0.41	0.44	0.38	0.32	0.33	0.35	0.34	0.35

Table S5. Study of the NFA Impact on the Physicochemical and Antiwear and Antiscuffing Properties of Polyurea Greases.

No.	Name of indicator	Base grease	Nano-functional additive								
			Amorphous silicon dioxide (KOVELOS 35/01T)			Calcium Carbonate ($CaCO_3$)			Calcium borate		
			The NFA concentration, %								
			1%	2%	3%	1%	2%	3%	1%	2%	3%
1	**Yield stress at 50°C, Pa**	**260**	360	250	190	460	400	360	360	390	370
2	Colloidal stability,% of oil emitted	**24.2**	18.3	18.1	19.2	16.3	18.1	19.3	18.7	19.4	18.6
3	Dropping point, ° C	**221**	> 250	> 250	> 250	> 250	> 250	> 250	> 250	> 250	> 250
4	Effective viscosity at minus 50ºC	**928**	1354	1453	1582	1324	1416	1635	1379	1349	1415
5	Lubricating properties on a four-ball friction machine under the temperature of (20 ± 5)°C:										
	- welding load, kgs	**112**	133	126	119	211	299	355	160	160	168
	- the wear scar, mm	**0.69**	0.56	0.75	0.79	0.72	0.66	0.64	0.61	0.48	0.45
